# Associations of sarcopenia and its defining components with cognitive function in community-dwelling oldest old

**DOI:** 10.1186/s12877-021-02190-1

**Published:** 2021-05-06

**Authors:** Anying Bai, Weihao Xu, Jing Sun, Juan Liu, Xinli Deng, Linna Wu, Xiao Zou, Jing Zuo, Lin Zou, Yunxia Liu, Hengge Xie, Xiaohong Zhang, Li Fan, Yixin Hu

**Affiliations:** 1 Peking University Health Science Centre, School of Public Health, Beijing, China; 2 Geriatric Health Care Department of The Second Medical Center & National Clinical Research Center for Geriatric Diseases, Chinese PLA General Hospital, Beijing, China; 3 Geriatric Department of Beijing North Hospital Of Ordnance Industry, Beijing, China; 4 Laboratory Department of The Second Medical Center & National Clinical Research Center for Geriatric Diseases, Chinese PLA General Hospital, Beijing, China; 5 Geriatric Emergency Department of The Second Medical Center & National Clinical Research Center for Geriatric Diseases, Chinese PLA General Hospital, Beijing, China; 6 Geriatric Cardiology Department of The Second Medical Center & National Clinical Research Center for Geriatric Diseases, Chinese PLA General Hospital, Beijing, China; 7 Neurology Department of The Second Medical Center & National Clinical Research Center for Geriatric Diseases, Chinese PLA General Hospital, Beijing, China

**Keywords:** Sarcopenia, Cognitive function, Oldest old, Muscle strength, Gait speed

## Abstract

**Background:**

This study aimed to investigate the associations of sarcopenia and its defining components with cognitive function in community-dwelling oldest old (over 80 years old) in China.

**Methods:**

Sarcopenia was diagnosed by the 2019 Asian Working Group for Sarcopenia (AWGS) criteria. Cognitive function was evaluated by the Montreal Cognitive Assessment (MoCA). Logistic and linear regression models were used to explore the associations of sarcopenia and its defining components with risk of mild cognitive impairment (MCI), and performance on multiple cognitive domains among 428 adults aged 80 years and older.

**Results:**

The overall prevalence of sarcopenia was 35.5%, with 40.34% for men and 32.14% for women. The prevalence of MCI was higher among sarcopenic oldest old than non-sarcopenic oldest old (28.95% vs. 17.39%, *p* = 0.005). Multivariate logistic regression analyses showed that sarcopenia [odds ratio (OR) = 1.86, 95% confidence interval (CI): 1.04–3.33], low handgrip strength (HS) [OR = 2.33, 95% CI: 1.40–3.87] and slow gait speed (GS) [OR = 2.31, 95% CI: 1.13–4.72] were significantly and independently associated with risk of MCI. Multivariate linear regression analyses showed that low HS was associated with worse performance in global cognitive function, visuospatial and executive function, naming and delayed recall.

**Conclusions:**

Sarcopenia, low HS and low GS was significantly associated with MCI in community-dwelling oldest old. The associations between sarcopenia and its defining components with different cognitive subdomains could be further explored in the future.

## Background

Sarcopenia, the age-related decline in skeletal muscle mass concomitant with decreased muscle function [1], has been formally recognized as a geriatric disease with an ICD-10-CM diagnosis code. In 2018, the European Working Group on Sarcopenia in Older People (EWGSOP2) updated the operational definition and clinical algorithm of sarcopenia by recommending using low muscle strength and low muscle mass to diagnose sarcopenia. Although the newest 2019 consensus of the Asian Working Group for Sarcopenia (AWGS) also contended that diagnosing sarcopenia required both the measurement of muscle mass, muscle strength and gait speed (GS) [2]. An increasing number of studies have indicated that single or multiple components, like muscle strength, is the key element in sarcopenia [3] in recent years. Growing evidence has shown that greater levels of handgrip strength (HS) is associated with lower risks of cardiovascular disease, all-cause and cardiovascular mortality, physical function, and frailty [4–6]. Therefore, muscle strength is emphasized as the trigger for further assessment and interventions by the introduction of the “probable sarcopenia” concept in the EWGSOP2 definition [7].

As the most prevalent cause of physical impairment, sarcopenia has repeatedly been associated with adverse outcomes such as frailty, hospitalization and increased mortality [7, 8]. Although some basic science and epidemiological studies have suggested a possible shared pathophysiology related to inflammatory markers and the hormonal pathway between sarcopenia and cognitive impairment [9, 10], evidence of this association among different populations remains controversial. The large variability was attributed to the methods of defining sarcopenia (based on single or multiple components), the age scope of the studied population and the usage of different detections for cognitive function. For instance, a prospective follow-up study with 555 older adults aged 85 years at baseline reported that poor HS predicted accelerated dependency in activities of daily living (ADL) and global cognitive decline using Mini-Mental State Examination (MMSE) in oldest old [11]. Studies among a population aged 60 years and over also found that low HS was associated with cognitive decline in information processing speed using measures of Digit Symbol Substitution Test (DSST) [12–14]. By contrast, another study included 14,775 Americans at least 50 years old reported that muscle strength capacity and cognitive function may parallel each other [15]. Results from the Tasmanian Study of Cognition and Gait (TASCOG) showed that decline in GS was associated with impairment in executive function, but not other cognitive domains among participants aged 60–85 years [16], while study on Canadians aged older than 65 years old showed no significant association between slower GS and poorer cognitive function detected by MMSE [17]. Moreover, no association was observed between decreased muscle mass and cognitive dysfunction detected by MMSE and the Peterson criteria after 7 y of follow-up in the EPIDOS-Toulouse group [18]. Although MMSE is the most widely used tool when assessing cognitive impairment, some studies report that the Montreal Cognitive Assessment (MoCA) is superior to the MMSE in differentiating mild cognitive impairment (MCI) patients from a healthy control group [19, 20].

Aging is an essential risk factor for sarcopenia and decline of cognitive function [21], and has been associated with a reduced activity tolerance attributed to changes in this skeletal muscle blood flow [22]. MCI is the abnormality of cognitive functions in populations matched for age and education levels, but without loss of functional abilities and skills in everyday social and occupational life [23], and it is associated with increased risk of developing dementia. In 2030, there are predicted to be 16.5 million people age 60 or older with dementia, 6.9 million males and 9.6 million females in China [24]. Identifying risk factors associated with MCI among the oldest old may help to develop early assessment tools for detection of MCI, enabling multi-domain lifestyle interventions at an early phase of dementia [25].

Previous studies showed that associations between handgrip strength, gait speed and cognition were not consistent among oldest-old individuals born in different decades [26], and the associations between blood pressure and cognition were contradictory among young and middle-aged subjects and oldest old subjects [27]. There is scarcity of information on the relationship between components of sarcopenia and different domains of cognitive function among the oldest old in China [28]. Therefore, in this study, we aimed to investigate the association of sarcopenia defined according to the updated 2019AWGS criteria and each of its defining components with performance of whole and multiple cognitive domains in community-dwelling oldest old (over 80 years old) in China.

## Methods

### Data and study participants

**Fig. 1:**
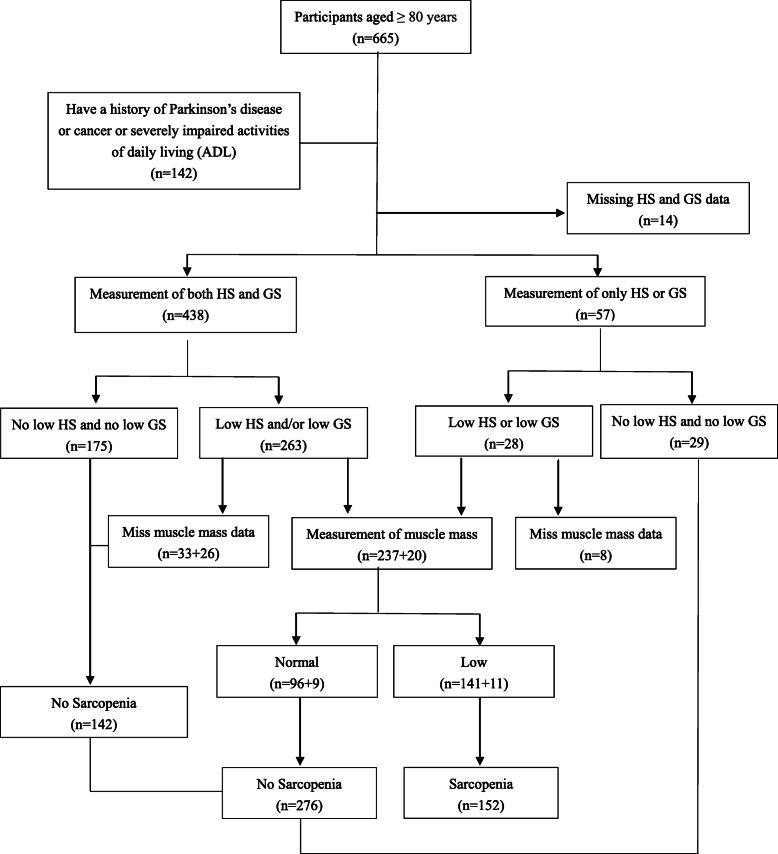
Flow diagram of the study population in the project

The present study is a cross-sectional analysis of individuals aged 80 years and older in the first wave of an ongoing longitudinal study, which is a group of elders living in a retirement community in Beijing. This study has been approved by the Research Ethics Committee of Chinese PLA General Hospital (Ethic number: S2018–102-02) and registered in Chinese Clinical Trial Register (ChiCTR1900022576). All methods in this study were performed in accordance with the relevant guidelines and regulations. A total of 665 oldest old who had electronic health records from long-run medical centers were recruited from 2018 to 2019 in one retirement community in Beijing, China. The interviews took place in their homes or in person at the community medical center, and information from their electronic health records were also collected. In the present study, people aged 80 years and over were included, those who revealed a history of Parkinson’s disease or cancer, or severely impaired ADL were excluded, and our final analysis included 428 participants with a written signed consent. More details on the inclusion process of studied population were provided in Fig. 1. All of the interviewers were well trained before the study.

### Sarcopenia

According to the AWGS criteria, sarcopenia was diagnosed if participants had low muscle mass plus low muscle strength or low physical performance [2]. The HS was measured twice for each hand using a digital handgrip dynamometer (JAMAR Co., Ltd., USA), and the maximum value of four tests was analyzed. Low muscle strength was defined as HS < 28 kg in men and < 18 kg in women. Body composition, including fat mass, fat free mass, and skeletal muscle mass, was measured using bioelectrical impedance analysis (BIA, InBody 270, Biospace Ltd., Seoul, Korea) by qualified research assistants. The appendicular skeletal muscle mass (ASM) was calculated as the sum of lean muscle mass in the arms and legs. According to the recommended method,^10^ skeletal muscle mass index (SMI) was defined as ASM divided by height squared. Low muscle mass was defined as SMI < 7.0 kg/m^2^ in men and < 5.7 kg/m^2^ in women based on the bioelectrical impedance analysis (BIA). Usual GS on a 6-m course was measured objectively, and was used to assess physical performance. Two trials were performed, and the shortest walking time was used to calculate GS and used in the analyses. Slow GS was defined as a gait speed of ≤1.0 m/s for both men and women.

### Cognitive function

Cognitive function was assessed using the Montreal Cognitive Assessment (MoCA), which was developed to enable earlier detection of mild cognitive impairment (MCI) [29]. Since there were no consistent criteria for MCI, the MCI diagnostic process in the current study was according guideline both from China [30] and American Academy of Neurology [31] as followed*:* 1) Historical report of subjective memory concerns or a change in cognition from the subject; 2) Objective evidence of impairment of cognition from MoCA; 3) Preservation of independence in functional abilities (do not have disability defined by ADL scores; 4) Not meeting the criteria for dementia by a dementia specialist. In this study, we diagnosed MCI based on the criteria considering both age and educational background of the studied participants in China and the cut-off of MCI among community-dwelling Chinese adults aged 80 years and over was 19 [32],

MoCA is a brief assessment of global cognitive function, visuospatial skills and executive function (0–5 scores), functions (0–5 scores), delayed recall memory (0–5 scores), attention (0–6 scores), naming (0–3 scores), language (0–3 scores), abstraction (0–2 scores), and orientation. (0–6 scores) [33]. The total score of MoCA ranges from 0 to 30, with higher scores indicating better cognitive function. Besides aging, education exerts a stronger influence than MCI diagnosis on variations in MoCA scores, and this is likely to adversely affect its test performance among poorly educated individuals. In this study, we diagnosed MCI based on the criteria considering both age and educational background of the studied participants in China. According to the validated age-specific cut-points in China, participants aged 80 years and older with total MoCA score ≤ 19 were categorized as MCI.

### Covariates

The covariates included in the analysis were sex, age, marital status (married vs. others including divorce, widowed, and never married), education, body mass index (BMI), smoke status (previous smoker vs. current smoker vs. never smoker), alcohol use, hypertension, diabetes, coronary artery disease conditions, depressive symptoms and low physical activity. Years of education was classified into three groups: below high school, high school and above high school. BMI was calculated as body weight divided by height squared and classified as underweight (< 18.5), normal (18.5–23.9), overweight (24.0–27.9), and obese (≥28.0) [34]. Physical function was assessed by HS, usual GS, time up and go test and five times sit-to-stand test. Depressive symptoms were assessed using the Korean version of 15-item geriatric depression scale (GDS-15), and participants whose scores were ≥ 8 were suspected to have a depressive tendency. Low physical activity was assessed by the total amount of walking time for exercise purposes. Women walking less than 120 min per week and men walking less than 150 min per week were defined as low physical activity [35].

### Statistical analysis

We described the socio-demographic, lifestyle, and health characteristics by AWGS-defined sarcopenia status. Normally distributed continuous variables were expressed as means ± SDs, and non-normally distributed continuous variables were expressed as median (inter - quartile range). Categorical variables were expressed as counts (percentages). Characteristics of participants according to sarcopenia status were compared using the analysis of variance test, Wilcoxon rank-sum test, chi-square test, and fisher’s exact test for normally distributed continuous variables, non-normally distributed continuous variables, categorical variables, and categorical variables with small expected values, respectively. Subsequently, we used logistic regression analysis to investigate the association between cognitive impairment and sarcopenia, including its defining components. We initially adjusted for socio-demographics (age, sex, education, and marital status) and subsequently added lifestyle and health characteristics. In addition, we identified the unadjusted and adjusted linear association of each component of the sarcopenia criteria (low muscle mass, low HS, and slow GS) with different domains of cognitive function as well as global cognition, separately. The adjustment of the models followed the same aforementioned pattern. All statistical tests were two-tailed, and *P*-values < 0.05 were considered statistically significant. All statistical analyses were performed using Stata version 14.0 (Stata, College Station, TX, USA).

## Results

### Subjects characteristics

Table 1 shows the characteristics of all participants according to sarcopenia status. A total of 152 (35.5%) participants were categorized as having sarcopenia according to the 2019 AWGS criteria. Participants with sarcopenia were significantly older (87.48 ± 3.68 VS 85.72 ± 3.34, *P* < 0.001), had worse nutrition assessment results (20.39% VS 7.61%, *P* < 0.001), higher prevalence of MCI (17.39% VS 28.95%, *P* = 0.005), lower HS (20.65 kg VS 23.8 kg, *P* < 0.001) and lower usual GS (0.79 m/s VS 0.93 m/s, *P* < 0.001).

**Table 1:** Participant’s Characteristics According to Sarcopenia by 2019 AWGS criteria (*N* = 428)

	Overall	Without Sarcopenia	With Sarcopenia	***P*** Value
	(***n*** = 428)	(***n*** = 276)	(***n*** = 152)	
**Age (years), mean ± SD**	86.34 ± 3.57	85.72 ± 3.34	87.48 ± 3.68	<.001
**Female,** ***n*** **(%)**	304 (60.32%)	171 (61.96%)	81 (53.29%)	0.081
**Higher Education (above high school), n (%)**	247 (49.40%)	139 (50.55%)	71 (47.02%)	0.78
**Married,** ***n*** **(%)**	298 (59.72%)	164 (59.42%)	93 (62.00%)	0.603
**BMI, (kg/m**^**2**^**)**	24.13 ± 4.61	25.12 ± 4.30	22.23 ± 2.82	<.001
**Obesity,** ***n*** **(%)**	108 (21.43%)	56 (20.29%)	5 (3.29%)	
**Low physical activity**	240 (47.62%)	126 (45.65%)	77 (50.66%)	0.927
**Smoke Status,** ***n*** **(%)**				0.79
**Previous smoker**	8 (1.60%)	5 (1.82%)	3 (1.97%)	
**Current smoker**	86 (17.20%)	45 (16.42%)	26 (17.11%)	
**Never smoker**	403 (80.60%)	221 (80.66%)	123 (80.92%)	
**Current Alcohol Use (> = 2–3 time/week),** ***n*** **(%)**	253 (51.52%)	132 (48.88%)	80 (53.34%)	0.353
**High comorbidity (Charlsen comorbidity index > 2)**	**115 (22.82%)**	58 (21.01%)	33 (21.71%)	0.866
**Hypertension,** ***n*** **(%)**	362 (73.43%)	204 (74.73%)	104 (70.75%)	0.379
**Diabetes mellitus,** ***n*** **(%)**	137 (27.57%)	74 (27.21%)	42 (27.81%)	0.893
**History of Coronary Artery Disease,** ***n*** **(%)**	264 (53.88%)	148 (54.61%)	73 (49.66%)	0.333
**Mild Cognitive Impairment (MCI),** ***n*** **(%)**	152 (35.51%)	48 (17.39%)	44 (28.95%)	0.005
**Depression,** ***n*** **(%)**	47 (9.33%)	23 (8.33%)	10 (6.58%)	0.646
**Poor Self-reported Health,** ***n*** **(%)**	176 (35.20%)	80 (28.99%)	57 (38.00%)	0.057
**MNA-SF(<=11)**	65 (12.90%)	21 (7.61%)	31 (20.39%)	<.001
**Physical function**				
**Handgrip Strength (kg),median (IQR)**	22.8 (19.2–27.7)	23.8 (20.6–29.1)	20.65 (17.6–25.1)	<.001
**Usual gait speed(m/s),median (IQR)**	0.85 (0.67–1.02)	0.93 (0.73–1.06)	0.79 (0.64–0.94)	<.001
**Time get up and go test (s),median (IQR)**	11.92 (9.87–15.09)	11.51 (9.71–14.23)	13.44 (10.37–16.32)	0.011
**Five times Sit-to-stand(s),median (IQR)**	14 (11.25–16.78)	13.57 (11.19–15.75)	15.00 (11.81–20.00)	0.011
**SMI, median (IQR)**				
**Appendicular Lean Mass index (kg/m**^**2**^**)**	6.32 (5.71–7.04)	6.62 (6.04–7.54)	5.60 (5.24–6.51)	0.425
**Appendicular Lean Mass to BMI ratio**	0.69 (0.58–0.82)	0.72 (0.62–0.83)	0.65 (0.54–0.78)	0.477

**Notes:** Values are means (± SD), median (inter - quartile range) or numbers (percentages). *BMI* body mass index, *GDS* Geriatric Depression Scale, *MNA* Mini Nutritional Assessment. *p*-values are based on the chi-square test, Fisher’s exact test, and the Mann–Whitney U test. Comorbidities were hypertension, myocardial infarction, dyslipidemia, diabetes mellitus, congestive heart failure, angina pectoris, peripheral vascular disease, cerebrovascular disease, osteoarthritis, rheumarthritis, osteoporosis, asthma, or chronic obstructive pulmonary disease, as diagnosed by a physician

Table 2 shows a summary of the cognition test scores in different cognitive subdomains measured by MoCA according to sarcopenia status defined by the AWGS. The sarcopenic individuals showed significantly lower scores of the global cognitive function (*P* = 0.017) and orientation (*P* = 0.011) than non-sarcopenic individuals.

**Table 2:** Neuropsychological test scores according to sarcopenia by AWGS criteria

	Overall (***n*** = 428)	Without Sarcopenia(***n*** = 276)	With Sarcopenia(***n*** = 152)	***P*** Value
**MoCA total score, median (IQR)**	23 (19–26)	23 (21–26)	22 (17–25)	**0.017**
**Visuospatial & Executive function, median (IQR)**	3 (2–5)	4 (2–5)	3 (2–5)	**0.056**
**Naming, median (IQR)**	3 (2–3)	3 (2–3)	3 (2–3)	0.759
**Attention, median (IQR)**	6 (4–6)	6 (5–6)	5 (4–6)	0.183
**Language, median (IQR)**	2 (1–2)	2 (1–2)	1 (1–2)	0.328
**Abstraction, median (IQR)**	2 (1–2)	2 (1–2)	1 (1–2)	0.232
**Delayed recall, median (IQR)**	2 (0–3)	2 (0–4)	2 (0–3)	**0.067**
**Orientation, median (IQR)**	6 (6–6)	6 (6–6)	6 (5–6)	**0.011**

### Association between sarcopenia, low muscle mass, low HS, low GS and MCI

The associations between sarcopenia, its defining components and risk of MCI are shown in Table 3. In the unadjusted model, sarcopenia (odds ratio (OR) = 1.93, 95% confidence interval (CI) = 1.21–3.09), low HS (OR = 2.41, 95% CI = 1.57–3.69) and low GS (OR = 1.82, 95% CI = 1.04–3.18) were independently and significantly associated with higher risk of MCI. After we adjusted for age, gender, race and education, there was no significant association between low GS and MCI. After leisure-time physical activity, ADL scores, smoking status, current alcohol intake, depression, diabetes, hypertension, previous history of coronary artery disease, and obesity were further adjusted, we found that compared with participants without sarcopenia, participants with sarcopenia were associated with higher risk of MCI (OR = 1.86, 95% CI = 1.04–3.33). Participants with low HS (OR = 2.33, 95% CI = 1.40–3.87) and low GS (OR = 2.33, 95% CI = 1.13–4.72) were also associated with MCI. Similarly, after all potential confounders were adjusted, we found that sarcopenia (OR = 3.26, 95% CI = 1.47–7.19), low HS (OR = 3.60, 95% CI = 1.81–7.16) and low GS (OR = 3.61, 95% CI = 1.36–9.63) were all independent risk factors of MCI.

**Table 3:** Associations between sarcopenia and its defining components with risk of MCI in older adults (*N* = 396)

Cognitive impairment	Sarcopenia	***P*** Value	Low musclemass	***P*** Value	Low muscle strength	***P*** Value	Low walking speed	***P*** Value
All participants	OR (95% CI)		OR (95% CI)		OR (95% CI)		OR (95% CI)	
**Unadjusted Model**	1.935 (1.211 ~ 3.093)	0.006	1.471 (0.943 ~ 2.295)	0.089	2.406 (1.567 ~ 3.692)	<.001	1.817 (1.037 ~ 3.181)	0.037
**Model 1**	1.996 (1.221 ~ 3.265)	0.006	1.482 (0.933 ~ 2.354)	0.095	2.233 (1.417 ~ 3.520)	0.001	1.736 (0.981 ~ 3.071)	0.058
**Model 2**	1.856 (1.035 ~ 3.327)	0.038	1.215 (0.684 ~ 2.156)	0.507	2.325 (1.396 ~ 3.873)	0.001	2.311 (1.131 ~ 4.722)	0.022

Note:Model 0: unadjusted model

Model 1: logistic regression adjusted for age, sex, race, and education

Model 2: logistic regression adjusted for age, sex, race, education, leisure-time physical activity, ADL scores, smoking status, current alcohol intake, depression, diabetes, hypertension, previous history of coronary artery disease, and obesity

### Association between sarcopenia, low muscle mass, low HS, low GS and performance on cognitive function

We further analyze the association of sarcopenia and its defining components with different domains of cognition assessed by MoCA (as shown in Table 4). Sarcopenia was associated with poorer performance in global cognitive function in crude analysis, although after adjustment for sociodemographic characteristics, psychophysical confounders and lifestyle, there was no association between sarcopenia and each domain of cognition. Low HS was associated with poorer performance in global cognitive function, visuospatial and executive function, naming and delayed recall after adjusting for confounders. There were no associations between low muscle mass, low GS and domains of cognition after adjustment.

**Table 4:** Association of Sarcopenia and Its Defining Components With Performance on Cognitive function by MoCA in Older Adults (*N* = 365)

Global cognitive function	Sarcopenia	*P* Value	Low muscle mass	*P* Value	Low muscle strength	*P* Value	Low walking speed	*P* Value
	β (95% CI)		β (95% CI)		β (95% CI)		β (95% CI)	
**Unadjusted Model**	−1.382(−2.488 ~ − 0.276)	0.014	−0.798(− 1.860 ~ 0.263)	0.140	−2.694(−3.717 ~ − 1.671)	<.001	− 1.165(− 2.268 ~ − 0.061)	0.039
**Model 1**	−1.306(− 2.422 ~ − 0.190)	0.022	− 0.693(− 1.747 ~ 0.361)	0.197	−2.431(− 3.476 ~ − 1.386)	<.001	−1.029(− 2.126 ~ 0.068)	0.066
**Model 2**	− 0.848(− 2.108 ~ 0.412)	0.186	− 0.195(− 1.426 ~ 1.036)	0.755	−2.125(− 3.217 ~ − 1.034)	0.022	− 0.806(− 1.977 ~ 0.364)	0.176
**Visuospatial & Executive function**	β (95% CI)	*P* Value	β (95% CI)	*P* Value	β (95% CI)	*P* Value	β (95% CI)	*P* Value
**Unadjusted Model**	− 0.329(− 0.663 ~ 0.006)	0.054	− 0.250(− 0.571 ~ 0.071)	0.127	− 0.782(− 1.115 ~ − 0.448)	<.001	− 0.404(− 0.741 ~ − 0.067)	0.019
**Model 1**	− 0.265(− 0.600 ~ 0.070)	0.12	− 0.199(− 0.515 ~ 0.118)	0.218	− 0.705(− 1.048 ~ − 0.363)	<.001	−0.369(− 0.702 ~ − 0.036)	0.03
**Model 2**	− 0.149(− 0.534 ~ 0.236)	0.446	−0.050(− 0.426 ~ 0.325)	0.792	− 0.775(− 1.139 ~ − 0.412)	<.001	− 0.059(− 0.252 ~ 0.133)	0.544
**Naming**	β (95% CI)	*P* Value	β (95% CI)	*P* Value	β (95% CI)	*P* Value	β (95% CI)	*P* Value
**Unadjusted Model**	−0.005(− 0.180 ~ 0.170)	0.952	− 0.024(− 0.195 ~ 0.146)	0.78	− 0.218(− 0.384 ~ − 0.053)	0.01	−0.046(− 0.222 ~ 0.130)	0.608
**Model 1**	−0.001(− 0.181 ~ 0.178)	0.988	− 0.020(− 0.193 ~ 0.153)	0.819	−0.225(− 0.398 ~ − 0.051)	0.011	−0.032(− 0.209 ~ 0.146)	0.725
**Model 2**	0.170(−0.031 ~ 0.372)	0.097	0.189(−0.011 ~ 0.389)	0.064	−0.214(− 0.396 ~ − 0.033)	0.021	0.056(− 0.116 ~ 0.228)	0.523
**Attention**	β (95% CI)	*P* Value	β (95% CI)	*P* Value	β (95% CI)	*P* Value	β (95% CI)	*P* Value
**Unadjusted Model**	−0.191(−0.477 ~ 0.096)	0.191	−0.262(− 0.547 ~ 0.023)	0.071	−0.414(− 0.687 ~ − 0.141)	0.003	−0.179(− 0.466 ~ 0.108)	0.22
**Model 1**	−0.177(− 0.463 ~ 0.109	0.223	−0.245(− 0.526 ~ 0.037)	0.088	−0.384(− 0.661 ~ − 0.107)	0.007	−0.148(− 0.429 ~ 0.133)	0.3
**Model 2**	−0.043(− 0.368 ~ 0.283)	0.797	−0.176(− 0.510 ~ 0.157)	0.298	−0.263(− 0.561 ~ 0.035)	0.083	−0.050(− 0.355 ~ 0.256)	0.75
**Language**	β (95% CI)	*P* Value	β (95% CI)	*P* Value	β (95% CI)	*P* Value	β (95% CI)	*P* Value
**Unadjusted Model**	−0.096(−0.276 ~ 0.084)	0.294	−0.129(− 0.300 ~ 0.041)	0.137	− 0.147(− 0.316 ~ 0.022)	0.087	−0.008(− 0.192 ~ 0.175)	0.982
**Model 1**	−0.042(− 0.222 ~ 0.137)	0.643	−0.081(− 0.249 ~ 0.086)	0.341	−0.031(− 0.202 ~ 0.139)	0.719	0.037(− 0.142 ~ 0.217)	0.683
**Model 2**	−0.065(− 0.271 ~ 0.142)	0.536	−0.121(− 0.320 ~ 0.077	0.23	0.014(− 0.168 ~ 0.196)	0.879	0.082–0.116 ~ 0.280	0.417
**Delayed recall**	β (95% CI)	*P* Value	β (95% CI)	*P* Value	β (95% CI)	*P* Value	β (95% CI)	*P* Value
**Unadjusted Model**	−0.338(−0.698 ~ 0.022)	0.066	−0.287(− 0.627 ~ 0.052)	0.097	− 0.923(− 1.260 ~ − 0.586)	<.001	−0.323(− 0.688 ~ 0.042)	0.083
**Model 1**	−0.278(− 0.643 ~ 0.086)	0.134	−0.232(− 0.571 ~ 0.107)	0.179	−0.826(− 1.168 ~ − 0.484)	<.001	−0.229(− 0.591 ~ 0.132)	0.213
**Model 2**	−0.282(− 0.704 ~ 0.140)	0.189	−0.219(− 0.626 ~ 0.187)	0.29	−0.853(− 1.214 ~ − 0.491)	<.001	−0.195(− 0.593 ~ 0.204)	0.338
**Abstraction**	β (95% CI)	*P* Value	β (95% CI)	*P* Value	β (95% CI)	*P* Value	β (95% CI)	*P* Value
**Unadjusted Model**	−0.087(−0.252 ~ 0.078)	0.229	−0.069(− 0.227 ~ 0.088)	0.386	− 0.131(− 0.287 ~ 0.025)	0.101	−0.184(− 0.351 ~ − 0.0177)	0.031
**Model 1**	−0.056(− 0.221 ~ 0.108)	0.501	−0.042(− 0.198 ~ 0.113)	0.591	−0.086(− 0.244 ~ 0.072)	0.285	−0.150-0.315 ~ 0.015	0.075
**Model 2**	−0.015(− 0.207 ~ 0.178)	0.881	− 0.004(− 0.192 ~ 0.184)	0.967	−0.094(− 0.264 ~ 0.076)	0.277	−0.177(− 0.360 ~ 0.007)	0.059
**Orientation**	β (95% CI)	*P* Value	β (95% CI)	*P* Value	β (95% CI)	*P* Value	β (95% CI)	*P* Value
**Unadjusted Model**	−0.198(−0.430 ~ 0.033)	0.093	−0.241(− 0.482 ~ 0.000)	0.05	− 0.143(− 0.377 ~ 0.090)	0.229	−0.015(− 0.250 ~ 0.219)	0.897
**Model 1**	−0.184(− 0.421 ~ 0.053)	0.128	−0.224(− 0.468 ~ 0.019)	0.071	−0.088(− 0.331 ~ 0.156)	0.48	−0.008(− 0.243 ~ 0.227)	0.946
**Model 2**	0.030(−0.238 ~ 0.298)	0.826	−0.012(− 0.298 ~ 0.273)	0.932	−0.033(− 0.291 ~ 0.224)	0.8	0.021(− 0.234 ~ 0.276)	0.873

Model 0: unadjusted model

Model 1: linear regression adjusted for age, sex, race, and education

Model 2: linear regression adjusted for age, sex, race, education, leisure-time physical activity, smoking status, current alcohol intake, depression, Diabetes mellitus, hypertension, previous history of coronary artery disease, and obesity

## Discussion

This study demonstrated that sarcopenia and its defining component HG and GS, were significantly associated with MCI in a sample of community-dwelling oldest old in China. This study also showed that low HS was associated with performance on global and several domains of cognitive function assessed by MoCA, including visuospatial and executive function, naming and delayed recall ability.

In our study, the prevalence of sarcopenia among community-dwelling oldest old was 35.5%, which was higher than people older than 60 (28.8%) in east China [36] and people over 50 (19.31%) in western China [37]. We found that AWGS-based sarcopenia was associated with higher risk of MCI. However, evidence from previous studies about the association between sarcopenia and cognitive function of older adults were controversial, which may be partly due to the different assessment tools of cognitive function and study population. A group study among British older men over 65 reported that no significant association was evidenced between FNIH-defined sarcopenia and mild or severe cognitive impairment assessed by MMSE [38]. Another cross-sectional analysis among 3025 women aged 75 years and older also demonstrated no significant associations between different operative sarcopenia definitions and cognitive impairment (measured by short portable mental status questionnaire) [39]. By comparison, our findings were consistent with previous studies in Asian populations [4, 37, 40]. In a cross-sectional analysis of 201 community-dwelling Korean women with the mean age of 74 years old, sarcopenia was inversely associated with MMSE and CES-D scores [41]. Also, in a prospective study with 131 adults aged 65 years and older in Japan, sarcopenia was founded to be an independent risk factor of cognitive deterioration assessed by MMSE during the 1-year study period [40]. However, the aforementioned studies were not conducted among the oldest old population and failed to examine the relationship between individual defining components of sarcopenia and different domains of cognitive function. Our study used MoCA to detect the mild stages of the cognitive impairment, which was able to differentiate between distinct clinical dementia syndromes at early stages of disease with high sensitivity and specificity [42]. Additionally, in this study, we found that sarcopenia was not associated with global and different domains of cognitive function after adjustment for multiple confounder. This result was conflicting, since most of the previous studies reported that sarcopenia-related cognitive impairment was mainly in the language and executive function using MMSE and Phonemic Verbal Fluency Test. (VFT) [38, 43] The discrepancy may be due to the smaller age range of our study population. Future longitudinal studies are still needed to clarify the association between sarcopenia and cognitive subdomains among populations in this age group.

This study found that low HS was not only associated with higher risk of MCI, but also associated with less scores in global cognitive function, visuospatial and executive function, and delayed recall in adjusted models, which indicated that low HS might be used as an early clinical screening tool for cognitive impairment among the oldest old population. Our findings are consistent with those of previous studies [11, 39, 44, 45]. Diana et.al found that poor HS is a predictor of accelerated cognitive decline using total scores of MMSE in the oldest old (> 85 years old) in Leiden [11]. Kim et.al also reported that baseline HS of 2378 Korean adults aged 65 or older was found to be positively associated with MMSE scores at baseline and over 8 years’ time [46]. Similar to our results, one cross-sectional analysis in Brazil also showed that low muscle strength was associated with poorer performance in all assessed cognitive domains [43], while Taekema et al. only found an association with MMSE changes and not with other cognitive variables, such as memory, attention, and processing speed [47]. Whether HS could be a predictive value for cognitive decline and its exact mechanism needed to be further explored in longitudinal studies.

Also, similar to previous findings [48–51], we found that low GS was associated with MCI after adjusting for all confounders. The associations between low GS and different domains of cognitive function were not fully studied in previous studies. Our study found that GS was positively associated with global cognitive function, visuospatial and executive function and abstraction in crude analysis. However, no associations were found after full adjustment, which was inconsistent with previous studies. Annika et al. reported that low GS was associated with impairment in executive function among community-dwelling older people with CI [52]. The heterogeneity of results may be due to differences in study populations and diagnosis criteria of low GS.

The associations between low skeletal muscle mass and incidence of MCI, as well as cognitive subdomains were also analyzed in our study. We found that low muscle mass was only associated with poorer performance in orientation in crude analysis, and no association was observed between low muscle mass and risk of MCI. Results among previous studies regarding the association between cognitive impairment and low skeletal muscle mass have been inconsistent: Won et al. found that skeletal muscle mass of elders older than 60 years old detected by bioelectrical impedance analysis (BIA) was linked with cognitive function [53], while Moon et al. showed that low muscle mass of Korean older adults was not associated with progression to cognitive impairment after 5 years of follow-up in the prospective study [54]. The cross-sectional analysis in Brazil also showed that low muscle mass was associated with poorer performance in the VFT [43] According to one systematic review, controversies among studies in the association of muscle mass with cognitive impairment were mainly due to differences of body composition devices [55], since the association might be stronger with use of BIA to measure body composition versus DEXA [56].

The possible mechanism between sarcopenia, low HS, low GS and cognitive function lies in that inflammations, oxidative stress, and hormonal changes share a common pathological role [57]. Several possible mechanisms may reinforce our conclusions that the association of sarcopenia and cognitive function is probably driven by alterations in muscle strength and not in muscle mass. First, muscle strength could reflect the change of brain-aging processes, such as the functioning of the central nervous system or white matter integrity [46]. Secondly, low HS and cognitive impairment may share common pathophysiological pathways such as systemic inflammation, insulin resistance and oxidative stress, all of which may contribute to both weak muscle strength and cognitive impairment [58]. Additionally, some studies also reported that changes in cognition and walking speed interact to predict future dementia [59]. It is thought that walking and cognition rely on similar brain regions, predominantly in the prefrontal cortex [60]. Neurodegeneration is a possible underlying mechanism linking declines in physical and cognitive function, with lower GS associated with changes in subcortical white matter and cortical gray matter volumes [61]. Another potential mechanism is neuroinflammation, which is thought to lead to impaired neuroplasticity in the brain areas controlling motor and cognitive function [60]. High concentrations of inflammatory markers are predictive of new-onset dementia and have also been implicated in mobility impairment [62].

The present study has many strengths. First, this is the first study to investigate the associations between sarcopenia and its defining components with different cognitive domains among Chinese community-dwelling oldest old, which provided extra support in addition to composite sarcopenia and global cognition function [63]. Our findings may have important implications in the early recognition of sarcopenia and cognitive impairment. Second, we used the MoCA scale as an early manifestation of MCI, which was more comprehensive and sensitive than other cognition measures with good test-retest reliability [64]. Third, our participants were a reliable group of community-dwelling oldest old coming from the real world, who had electronic health records from long-run medical centers. Our interview took place in these medical centers, which guaranteed reliability and were more representative of the real world.

There are several limitations in the present study. First, we used cross-sectional models so that causality could not be proved. Longitudinal studies will be required to illustrate the relationship between sarcopenia and MCI among adults older than 80 years old. Second, this study was done using a relatively small number of cases at one retirement community. However, our data can be considered as relatively valid because it was collected using standardized methods. Third, we only used MoCA to assess cognitive performance, which has no validated subscales in several cognitive domains. Therefore, other neuropsychological testing batteries are needed to have a more comprehensive assessment of cognitive abilities in future studies.

## Conclusions

The present results showed that sarcopenia was significantly associated with MCI among community-dwelling oldest old. Furthermore, our findings suggested that low HS may be useful to identify cognitive impairment, decreased visuospatial and executive function, naming and delayed recall at an earlier stage in non-disabled older adults living in the community. Additional longitudinal studies are needed to clarify associations between sarcopenia and its defining components and different domains of cognitive function.

## Data Availability

The datasets used and analysed in this study are available from the corresponding author on reasonable request.

## Abbreviations

HS: Handgrip strength
GS: Gait speed
MoCA: Montreal Cognitive Assessment
MCI: Mild Cognitive Impairment
EWGSOP: European Working Group on Sarcopenia in Older People
AWGS: Asian Working Group for Sarcopenia
